# I Choose to Opt-Out of Answering: Individual Differences in Giving Up Behaviour on Cognitive Tests

**DOI:** 10.3390/jintelligence10040086

**Published:** 2022-10-13

**Authors:** Marvin K. H. Law, Lazar Stankov, Sabina Kleitman

**Affiliations:** School of Psychology, University of Sydney, Sydney, NSW 2006, Australia

**Keywords:** giving up, metacognition, cognition, meta-reasoning, individual differences

## Abstract

Under the Meta-reasoning model, the process of giving up when a solution may not be feasible reflects an adaptive metacognitive strategy, where individuals opt-out of responding to mitigate error and resource costs. However, research is still needed to determine whether individuals systematically vary in this behaviour and if so, which variables it meaningfully relates with. The current study (N = 176) is the first to examine factorial stability in giving up tendencies and its relationships with on-task confidence, cognitive ability, decision-making predispositions, and academic performance. To measure giving up tendencies, participants completed three cognitive tests which allowed for opting out, thereby capturing giving up frequency within each test and its consistency across tests. Participants also completed five other cognitive tasks embedded with confidence ratings, and a decision-making styles questionnaire. Confirmatory factor analyses were conducted on all giving up, confidence, and accuracy variables, with a three-factor solution having the best fit (containing a giving up factor, confidence factor, and cognitive ability factor). Supporting the proposed adaptive nature of giving up tendencies, the giving up factor correlated positively with cognitive ability, rational decision making, and academic performance. This research establishes factorial stability in giving up tendencies and provides a foundation for further investigation into its role within Meta-reasoning theory.

## 1. Introduction

When faced with problems to solve, we balance our feelings of certainty before making the decision to continue solving or to give up. Depending on the strength of these feelings, the decision-making process can be quick and intuitive or slow and reflective (Ackerman et al. 2020). Ackerman and Thompson’s (2017) Meta-reasoning framework combines cognitive and metacognitive processes to explain how individuals solve problems and make decisions. Under this model, the decision to give up can occur at any point of the problem-solving process and has been posited to be an adaptive strategy to mitigate potential errors and resource costs (Payne and Duggan 2011). However, is there a trait-like construct surrounding giving up behaviour? Namely, do individuals vary systematically in these tendencies across different tasks? Furthermore, if so, what are the variables that share meaningful relationships with giving up tendencies? Using the Meta-reasoning model to establish hypotheses, this research examines these questions, contributing to further development of the model and the broader decision-making field of research.

According to the Meta-reasoning model, metacognitive processes both monitor and regulate reasoning during the problem-solving and decision-making process (Ackerman and Thompson 2017). These metacognitive processes allow individuals to gauge the effectiveness of their cognitive input whilst adjusting their cognitive resources based on the outcome and demand requirements (full description is available in Ackerman and Thompson 2017). One line of research has focussed on individual differences in cognitive and metacognitive traits. Findings from this research suggest that cognitive and metacognitive traits vary between individuals and can predict better decision making as well as academic achievement and life success (de Bruin et al. 2007; Parker and Fischhoff 2005; Toplak et al. 2017; West et al. 2008). For example, feelings of certainty, measured through on-task post-response confidence ratings, have been established as a robust metacognitive trait, confidence, which relates with life outcomes such as academic performance (Stankov 2013; Stankov et al. 2014; Stankov et al. 2015). However, one caveat of this research is that confidence ratings are based on chosen responses, where individuals must firstly decide on an answer to a problem before responding with a confidence rating that their chosen response is correct (Stankov et al. 2015). This caveat neglects problem-solving contexts where individuals may feel uncertain about a problem to the extent that they instead decide to give up on answering the question, choosing “I don’t know”. The current study seeks to determine whether there are robust individual differences in this type of giving up behaviour where the individual chooses to opt-out from the problem at hand.

Although there has been some investigation into opt-out decision making within experimental psychology (Ackerman 2014; Ackerman and Beller 2017; Goldsmith et al. 2014; Koriat and Goldsmith 1996; Payne and Duggan 2011; Weber and Brewer 2008; Weber and Perfect 2011) and animal psychology (Coutinho et al. 2015; Smith et al. 1997; Washburn et al. 2005), no research has yet been conducted on how individuals systematically vary in these decisions. Similarly, to the best of our knowledge, there is no research on how giving up tendencies relate to metacognitive traits, cognitive ability, and decision-making predispositions. Furthermore, the role of these tendencies on real-life performance outcomes have also not been investigated. Thus, this paper is a first proof of concept which aims to extend the current individual differences framework on decision making by including measures of opting out or giving up behaviour as adapted mainly from the animal metacognition literature. More specifically, we aim to examine the factorial structure of giving up tendencies, measured through the consistency in “giving up” behaviour within a task and across different tasks, and then compare it with the traits confidence, cognitive ability, decision-making predispositions, and academic performance.

### 1.1. Cognition and Metacognition

Although varying in some aspects, most theories of metacognition distinguish two groups of processes: (a) metacognitive monitoring, which monitors the allocation of cognitive resources; and (b) metacognitive control, which allocates cognitive resources (Ackerman and Thompson 2017, Nelson and Narens 1990). On-task, post-response confidence ratings (i.e., how confident are you that your answer is correct? Stankov et al. 2015) have been proposed to capture a key component of metacognitive self-monitoring, and have been vigorously investigated for decades, including their relationship with cognitive abilities and academic achievements. These feelings of confidence are theorised to accumulate throughout the problem-solving process, ultimately reaching a threshold, resulting in an individual to respond with a chosen answer (Koriat and Goldsmith 1996). Furthermore, this confidence can also determine a person’s willingness to commit their chosen answer for further scrutiny, such as submitting it for marking (Ackerman 2014; Koriat and Goldsmith 1996; Undorf et al. 2021).

Within the literature of individual differences, confidence has been shown to be a trait-like disposition, stable across differing contexts (see Stankov et al. 2015, for a review). Stemming from past decision-making research (Lichtenstein and Fischhoff 1977), a common paradigm is to measure confidence alongside cognitive ability within reasoning tasks using post-response confidence ratings (Ackerman 2014; Stankov and Lee 2008). In these studies, confidence and cognitive ability are measured by calculating overall mean confidence and accuracy, respectively, with both constructs being independent but positively correlated around the .3–.6 mark (Stankov and Lee 2008). The two constructs have been shown to predict real-life outcomes such as academic performance, with both contributing unique and significant variance (Neisser et al. 1996; Rohde and Thompson 2007; Stankov et al. 2012; Stankov et al. 2014). These findings highlight the combined role of cognitive and metacognitive capacities within real-life contexts.

However, this approach imposes the need for a forced-choice paradigm, where individuals must respond with an answer before rating their confidence. This paradigm ignores the possibility where an individual realises that they do not know the answer and thus prefers to opt-out from answering the item. The decision to give up, however, is proposed to be a key decision-making pathway which is driven by metacognitive monitoring. Importantly, by not providing an answer, the individual may be making a better choice for minimising error and resource costs.

The importance of examining strategically opting out of providing an answer to avoid errors (“giving up behaviour”) becomes particularly apparent when comparing research investigating metacognitive miscalibrations such as overconfidence and meta-ignorance (Blanchard et al. 2020; Buratti et al. 2013; Händel et al. 2020; Kleitman et al. 2019; Moore and Healy 2008; Schlösser et al. 2013). An individual who never gives up on any problem is bound to waste unnecessary time and cognitive resources. Similarly, within self-regulated learning, individuals who cannot give up on any learning goals may become overwhelmed (Boekaerts 1999). Acknowledging a lack of knowledge is particularly crucial in settings where ignorance can lead to further risk or harm (e.g., medical risk perception and health behaviours; Waters et al. 2013).

Despite its relevance to real-life problem solving and its inclusion within metacognitive models such as Ackerman and Thompson (2017)’s Meta-reasoning model, little research has investigated how people vary in this giving up behaviour and its relationship with other theoretically relevant variables. To address this gap in the literature, the current study examines individual differences in giving up behaviour in two important ways. Firstly, using a factor analytic approach, the current study aims to determine whether individuals give up systematically and in a generalisable manner across different cognitive tasks. If so, we will determine whether this behaviour defines a potential novel trait, which we will label as giving up. We will then relate this construct with the well-established trait confidence (Stankov et al. 2015), cognitive ability, decision-making predispositions, and academic performance.

### 1.2. Opting Out or Giving Up Behaviour

Although slight differences may exist in “opting out” and “giving up” terms, for the purpose of this research, we use both terms interchangeably to mean avoiding responding to a cognitive test item by indicating that they feel uncertain to continue. Existing research on “giving up” behaviour offers several different approaches to explain why an individual would choose to opt-out from solving a cognitive problem. Within this literature, some research has examined how individuals may give up on answering based on the perceived solvability of a problem and expended resources (Ackerman 2014; Payne and Duggan 2011). To explain giving up behaviour, Payne and Duggan (2011) proposed that individuals may give up by weighing the perceived probability of a question being solvable (*P*), the perceived gains of successfully answering the question (*G*), and finally the perceived costs of attempting the question (*C*) with the final guiding rule being *PG-C*. As *PG-C* approaches zero, participants become increasingly incentivised to give up on the question, rather than to solve it. The theory, however, does not stipulate whether the participants will apply this guiding rule consistently across different cognitive stimuli, thus, the factorial stability of this construct needs to be determined.

Following this guiding rule, we propose that the trait confidence may relate with individual differences in giving up tendencies, as confidence judgements would reflect the perceived capacity to solve a problem (*P*). Individuals with lower trait confidence would have lower values of *PG-C*, resulting in greater frequency of giving up. This dynamic between confidence and giving up would reflect the role of confidence ratings in driving decisions to withhold answers, a result which has been well established within experimental psychology (Koriat and Goldsmith 1996). However, as giving up tendencies are also determined by costs (*C*) and gains (*G*) associated with a problem, we do not expect giving up scores and confidence ratings to converge onto a single giving up/confidence factor. In contrast, we hypothesise that giving up scores and confidence ratings would converge onto two independent but negatively correlated giving up and confidence factors respectively. The negative correlation between the two would reflect the role of monitoring processes in driving control decisions, as presented within metacognitive models such as the Meta-reasoning framework (Ackerman and Thompson 2017).

The theoretical relationship between the proposed giving up factor and the cognitive ability factor is less clear and has never been investigated. Individuals with greater cognitive ability may give up less frequently simply due to their capacity to answer more problems correctly. However, we propose that individuals with greater cognitive ability should exhibit greater metacognitive awareness, by giving up on problems which are too difficult or resource intensive. Based on this latter premise, individuals with greater cognitive ability would be able to accurately follow the *PG-C* guideline to strategically give up, thereby minimising errors and resource costs. Conversely, individuals with low cognitive ability are more likely to deny or fail to follow *PG-C*, e.g., continuing solving despite the great cost of continued deliberation or responding with a likely incorrect response, despite having the option to opt-out. Thus, we hypothesise that giving up tendencies should relate positively with cognitive ability.

In addition to confidence and cognitive ability, decision-making styles may also relate with differences in giving up tendencies. Decision-making research has found that individuals vary in their approaches surrounding problem solving and making decisions, with five independent but interrelated styles of decision making (Scott and Bruce 1995). These styles include (1) rational, reflecting logical and methodical evaluations, (2) avoidant, reflecting delaying and avoiding decisions, (3) dependent, reflecting a need for advice and thoughts from others, (4) intuitive, making decisions based on feelings and instincts, and (5) spontaneous, reflecting making decisions quickly and impulsively without thinking deeply. Although some styles have been found to relate with metacognitive awareness, these decision-making predispositions have not been examined with giving up tendencies, nor with metacognitive trait confidence (Basu and Dixit 2022). Under the *PG-C* guideline, decision-making styles which prioritise strategic decision making or response avoidance should be associated with greater giving up tendencies. Based on this premise, we hypothesise that rational and avoidant decision-making styles should relate with greater giving up tendencies. The positive relationship with rational decision making will support the adaptive, strategic nature of giving up, while the positive relationship with avoidant decision making will suggest that giving up tendencies are reflective of response avoidance. It is unclear, however, how decision-making styles based on external dependence, minimal strategic thinking, and low risk consideration relate to giving up tendencies. As such, the remaining dependent, intuitive, and spontaneous decision-making styles are hypothesised to relate negatively or share no relationships with giving up tendencies. Ultimately, the primary aims for this study are to determine whether individual differences in giving up tendencies are systematic across tasks, and if so, whether these systematic tendencies would relate to confidence, cognitive ability as well as varying decision-making styles.

### 1.3. Academic Performance

The current study also aims to examine the role of giving up tendencies on real-world performance outcomes, i.e., academic performance. Within the individual differences literature, the role of both metacognition and cognition on academic performance is clear, with both confidence and cognitive ability factors positively relating to academic performance (Stankov et al. 2012, 2014). The error and resource cost mitigation strategy surrounding giving up tendencies can also be beneficial within academic settings, reflecting a capacity to know when to opt-out of answering. Thus, it is hypothesised that giving up tendencies would relate positively with academic performance.

### 1.4. Uncertainty–Monitoring Paradigm from Animal Metacognition

**Figure 1:**
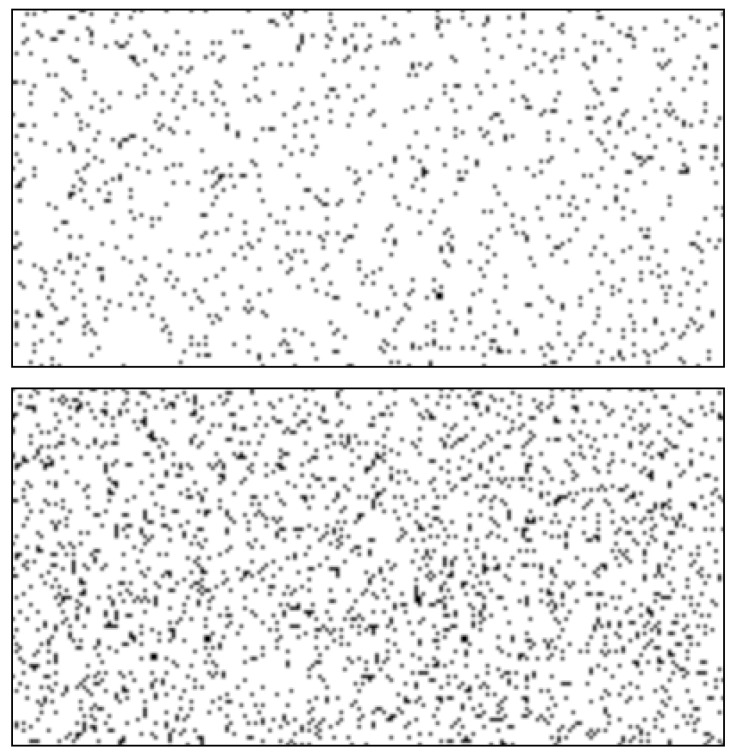
Example items from the SUD task. The box on the top is the least dense item, and the box on the bottom is the densest.

Existing experimental research has examined giving up behaviour within mostly animal participants, but also occasionally with humans, using a perceptual decision-making task known as the sparse–uncertain–dense (SUD) task (Beran et al. 2009; Coutinho et al. 2015; Smith et al. 1997, 2006; Washburn et al. 2005). The task was developed as an alternative approach to examining metacognitive monitoring, since traditional measures of self-report ratings (e.g., confidence ratings) were not feasible with animal participants (Smith et al. 1997). In this paradigm, participants are provided with boxes of varying degrees of pixel density and asked to determine whether they judged the box was sparse (S), dense (D) or whether they were uncertain (U) (example items shown below in Figure 1). To opt-out of responding, the participant must indicate that they feel uncertain, thus giving up on responding for the item and moving on to the next. Using 42 varying box items, participants were provided feedback for their S and D responses with either a 0.5 s whoop sound for correct responses or an 8 s buzz sound for incorrect responses. Uncertain responses were not given any feedback.

For animal participants, they would also be given food as a reward for correct responses. By running these items over multiple iterations, such animal research found the uncertain response to be used as a means for error monitoring, with animals (e.g., rhesus monkeys) and humans adaptively choosing the uncertain response for more difficult items (Smith et al. 1997). For both animals and humans, some individual differences emerged in the optimality of uncertainty responding both as the frequency as well as in the range of difficulty of choosing uncertain (Smith et al. 1997; Washburn et al. 2005).

Although the uncertainty response is a well-validated finding in animal research (Coutinho et al. 2015; Smith et al. 1997), it has yet to be compared systematically with confidence, cognitive ability, and decision-making predispositions as measured within the individual differences paradigm^1^. Adapting the SUD task to the current study, which uses human participants, aggregate uncertainty scores (i.e., frequency of indicating uncertainty and thus giving up) for each participant were computed for each test and for the relationships between these cumulative instances of “giving up” behaviour on different tests examined.

### 1.5. Aims and Hypotheses

In summary, we have two broad exploratory aims in the current study. The first aim is to examine whether there are robust individual differences in giving up behaviour across tasks. Secondly, we aim to investigate the relationships between giving up tendencies, and other theoretically important constructs, namely, the confidence trait, cognitive ability, and decision-making predispositions, as well as with academic performance. To the best of our knowledge, the current study is the first to examine the factorial structure of giving up tendencies, as well as its relationship with other key variables. We hypothesise that:

1. We postulate and compare four models: (1) a one-factor model with all performance accuracy, on-task confidence and giving up metrics defining one factor; (2) a two-factor model with performance accuracy defining one factor with confidence and giving up scores converging to define the second factor; (3) a three-factor model with a giving up factor, defined by all giving up scores across the three tasks, a confidence factor, defined by all confidence ratings, and a cognitive ability factor, defined by all performance accuracy variables; and finally, (4) a four-factor model with the broad accuracy factor splitting into two possible factors, accuracy for the tests with the embedded giving up scores and accuracy for the tests with the embedded confidence ratings. We expect that a three-factor solution would fit the data best.
2. The giving up factor will (a) correlate negatively with the confidence and (b) positively with the cognitive ability factors. It will also correlate positively with (c) rational and (d) avoidant decision-making styles, and negatively or not at all with (e) dependent, (f) intuitive, and (g) spontaneous decision-making styles. Finally, the giving up factor will (h) correlate positively with academic performance.

To determine the factorial structure of giving up tendencies, a Confirmatory Factor Analysis (CFA) was conducted on all giving up, confidence, and accuracy scores. Correlations between the subsequent factors and other variables (as specified above) were then examined.

## 2. Materials and Methods

### 2.1. Participants

The current study was conducted on 181 participants, 156 first year psychology students from the University of Sydney who participated for partial course credit using SONA recruitment, and 25 paid participants recruited through paid SONA at the University of Sydney. The paid sample were from a similar undergraduate cohort compared with those unpaid participants^2^.

Data cleaning: Data from five (one paid) participants were removed from any further analyses due to either non-attempts on most of the battery (item response times lower than one second for several measures) or non-completion of a substantial (>35%) number of measures. After filtering non-attempts for specific measures, except for academic performance (see below), some variables contained random missing values (together up to 6.8%). Expectation maximisation imputation was conducted on all missing values aside from academic performance using SPSS (IBM Corp 2020), as it provides reliable and unbiased parameter estimates (Graham et al. 2013). For the final analyses, there were 176 participants (131 Females, mean age = 20.31, SD = 3.90, age range = 17–59), which was sufficient to conduct factor analyses which required approximately 10–15 participants per variable within the model (Tabachnick and Fidell 2013).

### 2.2. Measures

To measure giving up, confidence, and cognitive ability, the current battery included eight cognitive tasks: three perception-based cognitive tasks (SUD, Cube Comparison and Visual Search tests), three traditional reasoning tasks (Esoteric Analogies, Advanced Raven’s Progressive Matrices, and Berlin Numeracy tests) and two heuristics and biases tasks (Cognitive Reflection and Applying Decision Rules tests). The battery also included the General Decision-Making Styles questionnaire, a measure of varying decision-making approaches (Scott and Bruce 1995). Other measures were included (e.g., personality, executive function tasks, additional heuristics and biases tasks), in conjunction with the measures for the current study, but were omitted as either being outside the scope of the focus of this study and/or having no systematic relationship with the variables of interest. Descriptive statistics, reliabilities, and correlation results are shown in Table A2 in Appendix A. Furthermore, the full data set and their descriptions are available on request from the corresponding author.

Giving up scores were measured within each of the three perception-based cognitive tasks, captured as the percentage of opt-out/giving up responses. To mitigate the possibility that giving up tendencies may converge simply due to being an artefact of response methodology, we varied the three tasks based on (1) punishment/reward mechanism, (2) the complexity/difficulty surrounding the cognitive processes involved, and (3) method of responding. *The punishment/reward system* for the SUD task was taken from the animal psychology literature and was based on time delays, i.e., participants were punished for incorrect responses by having to wait longer for the next item. As such, giving up decisions on this task would allow for avoiding punishment. In contrast, this type of punishment was not utilised for the Cube Comparison and Visual Search tasks. We also varied *the difficulty between tasks*, with the SUD task being the least difficult due to being adapted from an animal psychology paradigm. In comparison, the Cube Comparison Test was the most difficult, as it was from the Educational Testing Service (ETS) Kit of Factor Referenced Cognitive Tests and has been assumed to capture more complex visual processes associated with fluid reasoning (Nusbaum and Silvia 2011). Finally, the Visual Search task was judged to be in between the other two tasks in complexity and difficulty due to requiring emotional visual perception processes (further details available in Table 1). Finally, we varied *the response method* for the Visual Search Task compared to two other measures (see Table 1 in the Method section).

Confidence ratings were measured in the remaining five cognitive tasks, three traditional reasoning tasks and two heuristics and biases tasks. Confidence for each task was calculated by averaging post-response confidence ratings for each item, “how certain are you that your answer is correct?”: either from 0% to 100% or from 25% to 100%). These ratings were then averaged to determine each participants’ average confidence. Finally, performance accuracy was also captured for all cognitive tasks, measured by the percentage of correct responses.

### 2.3. Procedure

The study was conducted via computers within an experimental university lab, with participants completing all tasks and a demographic questionnaire measuring variables including age, sex, and English as a first language in a 2 h block. To account for counterbalancing, participants completed one of three different test blocks with differing task orders. Due to the long protocol, participants were encouraged and were able to take short breaks during the testing session.

## 3. Results

To determine the factorial structure of giving up tendencies, after examining the pattern of correlations, we conducted a confirmatory factor analysis (CFA; maximum likelihood with geomin rotation) on giving up variables (SUD, Cube Comparisons, and Visual Search tasks), confidence variables (Esoteric Analogies, Berlin Numeracy, Advanced Raven’s Progressive Matrices, Cognitive Reflection Test and Applying Decision Rules tasks), and performance accuracy scores from all eight tasks.

### 3.1. Descriptive Statistics, Reliabilities, and Correlations between Giving Up, Confidence, Performance Accuracy and Life Outcome Measures

Descriptive statistics, reliabilities, and correlations between all metacognitive and cognitive variables are provided in Table 2 below. The descriptive statistics and reliabilities for all remaining variables can be found in Appendix A (Table A1).

**Figure 2:**
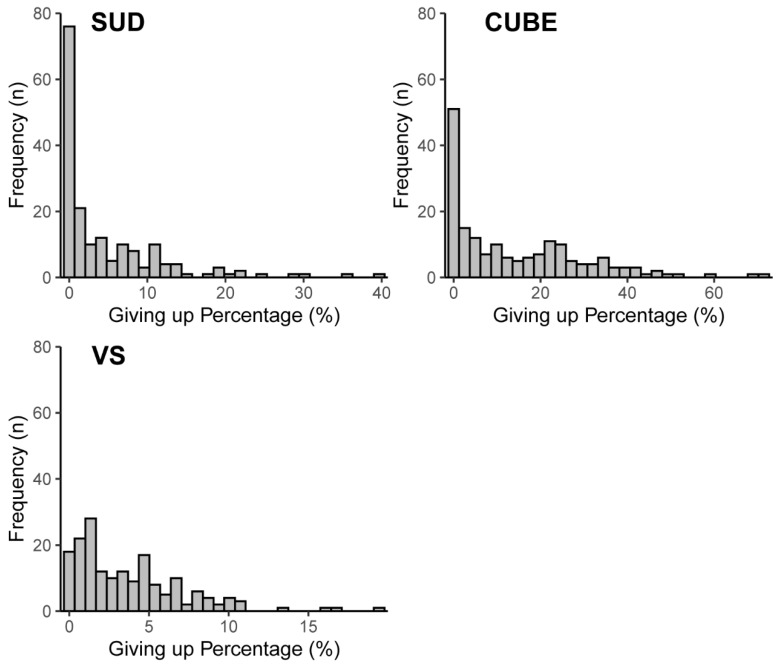
Frequency histograms for the giving up scores in the SUD, CUBE, and Visual Search tasks.

Internal consistency ranged from acceptable to excellent (.64 to .94) for all metacognitive and cognitive variables (Cronbach 1951), except for BNT accuracy (.55). Average giving up scores were generally low and varied substantially between the three tasks, ranging from 3.73% to 14.02%. The average frequency of giving up decisions for all tasks ranged between five and seven times. Despite high positive skew on giving up scores (see Figure 2 below), only five participants (less than 3%) had never given up on any task. Furthermore, 35%, 43% and 54% of participants had given up at least five times on the SUD, Cube Comparisons, and Visual Search tasks. respectively. These results suggest that despite having low average giving up scores, the current study captured a reasonable distribution of opt-out behaviour, and these tendencies were systematic with the internal consistency estimates for giving up tendencies ranging between .78 and .94. Despite having an added opt-out option, performance accuracy for the Visual Search task in the current study (~71%) was similar compared with the same task in other studies where no opt-out option was provided (~72%, Law et al. 2018). Accuracy scores between the three tasks embedded with opt-out options also varied. Confirming our expectations, accuracy was highest for the SUD task (87%), being 12% to 16% higher than the other two tasks. Although we had expected the Cube Comparisons to be more difficult than the Visual Search task, their accuracy scores were similar, being 75% and 71% respectively.

To examine potential item order effects, giving up rates were examined based on the presentation order of each item for the three tasks (randomised for the SUD and non-randomised for the other tasks). Although rates of giving up appeared to be relatively stable across item order, early to middle items for the SUD and Visual Search tasks appeared to have greater marked differences in giving up rate between proximal items (e.g., giving up rates for the Visual Search task was 8% for item 4 but 3% for item 5). In comparison, items shown later in the task exhibited more stable giving up rates (e.g., items 11 to 20 of the Visual Search task have giving up rates between 2% and 4%). This behavioural pattern was not observed for the Cube Comparison task.

Between the giving up variables, two of the three correlations were positive and significant: .21 and .32. Furthermore, there was a positive manifold between all confidence ratings (correlations range: .29 to .61). Finally, most performance accuracy variables significantly and positively correlated with each other aside from SUD Task accuracy and Visual Search Task accuracy. SUD Task accuracy did not correlate significantly with performance accuracy from any other task. Visual Search accuracy correlated positively, albeit weakly with all other performance accuracy variables, with only two correlations being significant: EAT accuracy (*r* = .15, *p* < .05) and BNT accuracy (*r* = .18, *p* < .05).

Giving up scores only correlated significantly with eight variables based on confidence ratings and performance accuracy. Cube Comparisons giving up score shared significant negative correlation with all confidence variables (ranging between −.29 and −.16). Furthermore, SUD Task giving up score shared significant positive correlations with BNT and SUD Task accuracy (.16 and .20 respectively). Finally, Visual Search giving up score correlated significantly and positively with both CRT and SUD Task accuracy. All performance accuracy variables besides SUD Task and Visual Search Task accuracies had significant and positive correlations with all confidence variables (correlations range: .18 to .69). In contrast, SUD Task and Visual Search Task performance accuracy did not correlate significantly with any of the confidence variables. These relationships are summarised by the results of the Confirmatory Factor Analysis below.

### 3.2. Confirmatory Factor Analysis (CFA) of Cognitive and Metacognitive Variables Using Maximum-Likelihood Method

To test the factorial structure underlying giving up, confidence, and performance accuracy variables, five CFAs solutions were conducted with the Maximum Likelihood method and geomin rotation using the lavaan package within the R software (R Core Team 2022; Rosseel 2012) (descriptions and fit indices of all models can be found in Table 3 below). Using fit indices cut-off conventions from the literature, model fit was defined to be acceptable if Standardized Root Mean Squared Residual (SRMR) ≤ .08, Root Mean Square Error of Approximation (RMSEA) ≤ .06, Comparative Fit Index (CFI) ≥ .95, and Tucker–Lewis Index (TLI) ≥ .95 (Brown 2015; Hu and Bentler 1999).

Firstly, an initial one-factor model was conducted with all variables loading onto a single factor with the model fit being poor (Brown 2015; Hu and Bentler 1999). Subsequently, a two-factor model (capturing a giving up/confidence factor and a cognitive ability factor) and a three-factor model (capturing a giving up factor, a confidence factor, and a cognitive ability factor) were conducted to determine the factorial independence of giving up tendencies. For the two-factor model, all giving up variables and confidence ratings defined a single giving up/confidence factor whilst all accuracy scores defined a cognitive ability factor, with both factors being allowed to covary. In contrast, the three-factor model had all giving up variables define a giving up factor, all confidence ratings define a confidence factor, and all accuracy scores define a cognitive ability factor, with all factors covarying freely. Comparing differences in χ^2^ statistics indicated that the two-factor solution was an improvement compared to the one-factor solution (χ^2^ diff = 77.84, *p* < .001); however, the fit indices remained poor (Brown 2015; Hu and Bentler 1999). Subsequently, the three-factor solution demonstrated a significant improvement over the two-factor solution (χ^2^ diff = 25.54, *p* < .001) with better fit indices; however, the overall model fit was still poor. Thus, a four-factor solution was conducted with the broad accuracy factor being split into two possible factors: accuracy for the tests with the embedded giving up scores and accuracy for the tests with the embedded confidence scores. This model, however, showed no significant improvement over the three-factor-model (χ^2^ diff = 6.88, *p* = .08), suggesting that the three-factor model fit the data best; however, the low Tucker–Lewis Index suggested that some important paths may have been missing. Thus, we examined and incorporated several minor modifications.

**Figure 3:**
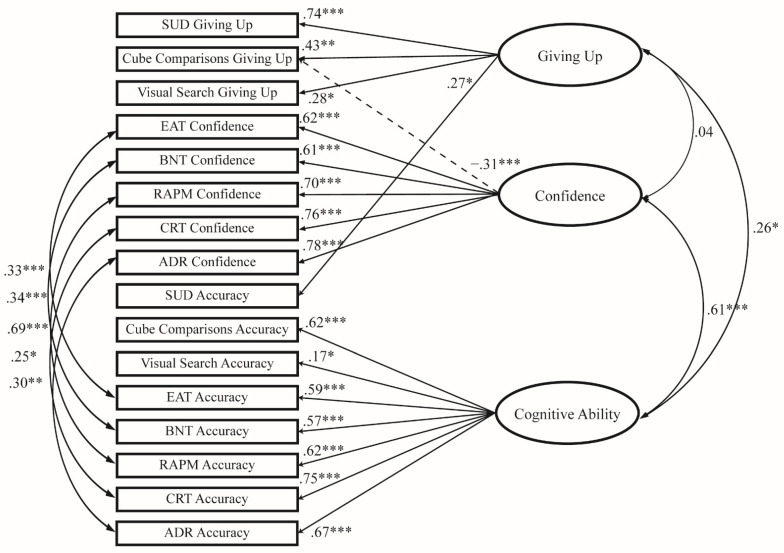
Summary path model of the final three-factor CFA model with standardised regression and correlation coefficients * *p* < .05, ** *p* < .01, *** *p* < .001.

Firstly, following prior literature, given the strong relationship between confidence ratings and accuracy, residuals between accuracy and confidence from the same measures were allowed to covary (Jackson et al. 2016; Kleitman et al. 2019). Although there are concerns about allowing residuals to correlate due to violating the assumption of independence of residuals, the correlations between the residuals for the different scores within the same test are acceptable, as they capture non-random disturbances due to the nature of the test (Brown 2015). Cube Comparisons giving up score was also allowed to load onto the confidence factor, as it correlated negatively with all confidence ratings. Finally, SUD accuracy was allowed to load on the giving up factor instead of the cognitive ability factor given that its only significant (albeit weak) correlations were with giving up scores. The final modified model had acceptable fit based on cut-off fit conventions (Brown 2015; Hu and Bentler 1999). Although loadings varied in sizes, they all were statistically significant (*p* < .05). A path diagram summarising the final model is shown in Figure 3 below.

The three factors in the final model were interpreted as:

Factor 1: Giving up—this factor was defined by all giving up variables as well as with a positive loading from SUD accuracy. This factor is indicative of robust individual differences in giving up tendencies.

Factor 2: Confidence—this factor was defined by all confidence variables, as well as negatively defined by giving up score on the Cube Comparisons task. This factor is clearly indicative of the metacognitive confidence trait. The negative loading from Cube Comparisons giving up scores indicates that high-confidence individuals may be related to giving up tendencies in some contexts.

Factor 3: Cognitive ability—this factor was defined by all accuracy variables except for SUD accuracy. This factor is indicative of cognitive ability. The low loading from Visual Search accuracy on this cognitive ability factor indicates that this factor is more aligned with fluid and crystallised intelligence, rather than perceptual ability. However, Cube Comparisons accuracy had a strong positive loading on this factor, supporting the general nature of this factor which included a test embedded with giving up response options.

The giving up factor did not correlate significantly with the confidence factor (*r* = .04). However, supporting Hypothesis 2b, the giving up factor correlated significantly and positively with the cognitive ability factor (*r* = .26). Finally, replicating numerous studies (e.g., Stankov and Lee 2008; Stankov et al. 2015), the confidence factor correlated significantly, positively, and strongly with the cognitive ability factor (*r* = .61).

### 3.3. Comparisons between Giving Up, Confidence, and Cognitive Ability Factors with All Other Variables

To compare the resultant factors with other variables, factor scores were estimated using the Bartlett method within the lavaan package (Rosseel 2012). Subsequently, *t* tests and correlations were conducted to compare the three extracted factors (giving up, confidence, cognitive ability) with decision-making styles and academic performance. Pairwise comparisons between sex and English as a first language indicated no significant differences in giving up factor scores for either variable. However, females had significantly lower confidence (mean difference = −4.54) and cognitive ability (mean difference = −4.79) than males, t(174) = −3.60, *p* < .001 and t(174) = −3.07, *p* < .001, respectively. Correlations between the three factors resulted from the CFA, and other variables are shown in Table 4 below.

Seven significant correlations were found between the three factors and other variables. Supporting Hypothesis 2c and 2h, the giving up factor correlated positively with both rational decision-making style and academic performance, albeit weakly. The confidence factor also correlated positively and weakly with rational decision-making style and academic performance, and negatively and weakly with an avoidant decision-making style. Finally, the cognitive ability factor correlated negatively and weakly with intuitive decision-making style, and positively and moderately with academic performance.

## 4. Discussion

The current research is a novel study which is a proof of concept into systematic individual differences in giving up tendencies, and their relationships with confidence, cognitive ability traits, decision-making predispositions, and academic performance. The decision to give up has been posited to be an adaptive strategy to mitigate potential errors and resource costs (Ackerman 2014; Koriat and Goldsmith 1996; Payne and Duggan 2011). However, to the best of our knowledge, previous research has not examined whether individuals vary systematically in the use of this strategy, and if so, what other psychological constructs might hold meaningful relationships with these tendencies. The current study aimed to investigate these two questions. Following Payne and Duggan’s (2011) *PG-C* model, we proposed that confidence, cognitive ability traits, decision-making predispositions, and academic performance should all relate with systematic giving up tendencies.

To examine these premises, the current study employed eight cognitive tasks, three embedded with an opt-out option, and five embedded with post-response confidence ratings. CFA were conducted on all giving up, confidence, and accuracy variables to determine the factorial structure of giving up tendencies. Finally, *t* tests and correlations were conducted between the resultant factor scores with all other variables.

### 4.1. Are Giving Up Tendencies Systematic?

Our CFA results support the premise that giving up tendencies are systematic across tasks, with the best fitting solution having three factors: all giving up scores converging onto a novel giving up factor, and confidence ratings and performance accuracies converging onto two separate confidence and cognitive ability factors, respectively. However, to improve the model’s fit, we incorporated two post hoc cross-loadings. Although post hoc, these cross-loadings make sense and can potentially inform future studies. Firstly, SUD accuracy loaded positively and weakly onto the giving up factor. This cross-loading can be explained by the strategic role of giving up towards minimising potential errors embedded in the test’s scoring system. Individuals who give up more often are likely to have higher accuracy scores within the same task due to opting out of more difficult questions. Thus, this cross-loading demonstrates the error mitigation strategy of giving up, with participants having increased accuracy by opting out and reducing potential errors. Secondly, giving up score on the Cube Comparisons task loaded negatively onto the confidence factor. Although the giving up factor did not correlate significantly with the confidence factor, this cross-loading suggests that giving up tendencies may be negatively related to confidence within certain task contexts. As such, we suggest further studies comparing giving up tendencies and confidence from similar or the same tasks. Despite these two significant cross-loadings, the CFA solution has robust fit, establishing the novel giving up factor, which is representative of systematic giving up tendencies. Within the tests, participants also exhibit high internal consistency in their giving up responses, with Cronbach’s alpha estimates ranging between .78 and .94. Altogether, although largely exploratory, these results provide strong support that giving up tendencies are systematic within and across tasks.

### 4.2. Correlations with Other Variables

We had expected that the giving up factor would correlate significantly with confidence, cognitive ability, decision-making predispositions, and academic performance. However, there were only three significant and positive, though weak, correlations, namely, with cognitive ability, rational decision making, and academic performance. Normative expectations suggest that individuals who give up more often would likely be those who are less capable. However, our findings support an alternative, and possibly strategic nature of giving up, such that individuals who give up are those with greater cognitive ability and are doing so based on a rational and logical approach. Decisions to give up on responding are often viewed negatively, particularly when frequent (Grice 1975; Goldsmith 2016). Thus, rational decision makers and those who are more intelligent may be able to judge the benefits of giving up as an error mitigation strategy, resulting in more frequent opt-out decisions. The adaptive benefits of this approach can ultimately allow individuals to perform more effectively within academic settings. Although promising, we acknowledge that these correlations are weak and need to be replicated in future studies.

Despite the strong theoretical relationship, there was no significant correlation between the confidence and giving up factors, suggesting that those with low confidence did not opt to give up more often. One explanation for this non-significant relationship may be due to the strategic nature of giving up. As giving up is posed as a strategy to avoid potential errors and costs, individuals who give up more may be those who are error and cost avoidant, rather than those with low confidence. Namely, some individuals may still prefer to avoid incorrect responses despite having high trait confidence. In contrast, some individuals with low trait confidence may prefer to respond with an answer, despite having a lower perceived likelihood of answering correctly. This preference may explain participants choosing low-confidence responses even when given the opportunity to opt-out (Ackerman 2014). However, we note that in experimental research, opt-out decisions have been found to be associated with low-confidence judgements even in conditions where alternatives to opting-out are permitted such as seeking help or providing less informative answers (Undorf et al. 2021; Weber and Brewer 2008). Thus, our current findings should be replicated and extended within experimental paradigms, to determine the nature of its relationship, however, focusing between trait confidence and the novel giving up factor.

Instead of trait confidence, individual differences reflective of other metacognitive judgements may drive giving up tendencies. One potential avenue for investigation is the initial judgements of solvability (iJOS), the perceived judgment of being able to solve a problem after initial evaluation (Ackerman and Thompson 2017). Unlike confidence ratings, IJOS are made early on in the problem-solving process and have been shown to predict later decision-making behaviour, e.g., solving time and post-response confidence (Lauterman and Ackerman 2019). They have also been suggested to control decisions to give up and time until giving up (Ackerman and Thompson 2017; Lauterman and Ackerman 2019; Payne and Duggan 2011). Thus, we suggest future studies to examine other metacognitive processes, e.g., solvability judgments, which may relate to giving up behaviour.

### 4.3. Limitations and Future Direction

There were several limitations within the current study. Firstly, our results were limited by low rates of giving up decisions. These low rates of opt-out decision have been seen in previous studies, suggesting that decisions to opt-out are inherently low even within other contexts (Ackerman 2014; Hanczakowski et al. 2013; Washburn et al. 2005). Furthermore, as mentioned before, these low rates may be reflective of the large number of items used in our tasks, with the average giving up frequency ranging from five to seven for all three tasks. Thus, we propose that the current distribution of giving up rates, although low, is reasonable in examining individual differences in a behaviour that is generally avoided. Nevertheless, increasing rates of giving up behaviours may provide greater spread and individual differences in the behaviour. The current study measured giving up rates using reasoning tasks which were easier than standard reasoning tasks used to measure cognitive ability. Thus, future studies can increase giving up rates by implementing tasks with greater task difficulty. Additionally, giving up rates may also increase with greater costs associated with incorrect responses. As such, tasks embedded with greater punishments for incorrect answers are also more likely to incentivise giving up decisions. Furthermore, we also suggest that future studies should investigate potential item order effects within tasks using the opt-out procedure.

Another limitation was that only frequency of giving up was examined within our study. Existing models of giving up (Ackerman and Thompson 2017; Payne and Duggan 2011) suggest that there are varying stages during problem solving where an individual may decide to give up, with differing motivations. For example, the decision to give up may come immediately after seeing a problem if it is judged to be unsolvable, or it may come after deliberation if solving efforts are unfruitful. Predispositions towards these divergent giving up decisions may also be a source of individual differences worth investigation. Thus, we suggest future studies to examine giving up at different stages of problem solving, as well as individual differences in varying giving up approaches.

As the current study was a preliminary examination into giving up behaviour, its investigation into task motivation was also limited, with only the SUD task having a reward/punishment system. With no incentives for high task performance and no punishment for choosing to give up, participants may have chosen to give up due to low task motivation. However, this is an unlikely explanation, as giving up scores were greatest for the Cube Comparisons task where participants were not punished for responding incorrectly. Furthermore, frequencies of giving up were similar across all giving up tasks. Nevertheless, we recommend that future studies should incorporate more substantial reward/punishment paradigms as well as control for task motivation.

The study was also limited by differences in the cognitive tasks measured. There were substantial differences in average accuracy scores across the eight employed cognitive tasks (42% to 87%), and this could have affected the results given the known hard–easy effect, where individuals overestimate their success towards hard tasks and underestimate success towards easy tasks (Suantak et al. 1996). In particular, the tasks embedded with giving up had higher accuracy when compared with the tasks embedded with confidence ratings. These differences may have been due to the role of giving up in increasing output-bound accuracy, where participants can choose to give up for more difficult items, leaving only the easier items to be calculated for accuracy (Koriat and Goldsmith 1996). To address these limitations, we suggest further studies to compare giving up and confidence from similar tasks with the same range of difficulty. We must note, however, that further studies in our laboratory have utilised the mentioned modifications to the research design and have replicated these findings.

Our current study was further limited by the length of the protocol. Due to the extensive battery required to investigate our aims, testing sessions lasted approximately two hours, undoubtedly causing fatigue. Although our protocol was counter-balanced to control for fatigue, future research would benefit by using these findings to develop more concise studies. As a further limitation, we acknowledge that behaviour can vary based on environmental factors such as time constraints and task demands outside of the experiment. These factors are difficult to control for within single-session experiments such as the current study, and we recommend that future studies investigate the stability of individual differences in opt-out behaviour using longitudinal experiment protocols.

## 5. Conclusions

The current study is a novel investigation into individual differences in giving up behaviour, more broadly examining the existence of the metacognitive control trait, giving up. CFA revealed a best fitting three-factor solution, with giving up, confidence, and performance accuracy variables loading respectively onto three separate and independent factors. These findings support the premise that individuals give up systematically across different tasks as a metacognitive strategy to minimise errors and resource costs during problem solving. This is a premise that serves as a much-needed foundation for future investigations. Importantly, the study also found significant positive, albeit weak, relationships between this newly established giving up factor with cognitive ability, rational decision making, and academic performance, suggesting that more intelligent and rational decision makers may be more predisposed to give up as an adaptive strategy. These tendencies may also benefit academic outcomes. Future research should investigate both the characteristics of this novel giving up factor and its potential underlying processes to further understand its metacognitive nature and its role in learning, cognitive processing, and decision making.

## Figures and Tables

**Table 1:** Summary of measures used in this study.

Measure	Example Item	Reliability from Previous Studies
**Cognitive Tasks with embedded giving up options**		
*Sparse-Uncertainty-Dense (SUD) Task—Beran et al. (2009)*. A visual processing task which was developed within animal psychology research to examine metacognition in animals and humans (Beran et al. 2009; Smith et al. 2006). For this task, participants are shown pixel density boxes and were asked to decide whether the number of pixels in the box constitutes as sparse or dense with correct responses eliciting rewards and incorrect responses eliciting punishments. The most sparse and dense items were given to participants as practise prior to the task. For the current study, a point system and item time delays were used as reward and punishment incentives with correct responses leading to a point increase and time delays between items of one second whilst incorrect responses had a point reduction and a time delay of eight seconds ^a^. To measure giving up, participants were given a third option to select that they are uncertain about the answer, which will skip over the current item, eliciting no point change and a time delay of two seconds. Following prior studies, pixel density plots were 200 × 100 pixels in area with pixel densities ranging from 1085 to 2255 with a 1.8% increase in pixel density per level (Beran et al. 2009). Ultimately, 42 levels were constructed with the first 21 defined as sparse and the latter 21 defined as dense. These pixel boxes were constructed using a pixel density randomisation script in JavaScript (source code available by request). Participants were shown each level three times over the task. Overall accuracy and giving up scores were measured for this task, with accuracy being the percentage of correct responses out of all attempted items and giving up scores, being the percentage of uncertain responses out of all items (126—three repetitions of the 42 items).	see Figure 1	-
*Cube Comparisons—Ekstrom et al. (1976)*. The Cube Comparisons Task is a measure of visual processing from the ETS’s Kit of Factor Referenced Cognitive Tests with 42 items. For each item, participants are shown three faces of two cubes and must decide if they think that the cubes could potentially be the same cube or are definitely different. Similar to the SUD task, a third “uncertain” option was added for when participants were uncertain about answering. Prior to the task, participants were shown some practise items with feedback. Overall accuracy and giving up scores were calculated in the same way as that in the SUD task. For this task, there was no feedback nor reward/punishment for responding.	See Ekstrom et al. (1976) and Figure A1 in the Appendix A.	Original measure: .84 (Kozhevnikov and Hegarty 2001).
*Visual Search for Faces with Corresponding Emotion Expressions of Different Intensity—Short Form—Wilhelm et al. (2014)*. This task is a short 20-item measure of emotion perception. For each of the twenty items, participants are shown 9 different faces of the same individual in a 3 × 3 grid, with each face displaying a certain emotion. Participants must discern which of the faces do not express the same emotion as the majority of the faces. Adapting to measure giving up, an “uncertain” option was provided for each of the faces in the items, with participants being allowed to choose uncertain rather than make a decision on whether the face was part of the majority emotion or not. As a result, participants were not allowed to select uncertain and make a decision on the same face. Participants were shown an example item before the task. Following prior research, accuracy on the task was measured using Unbiased Hit Rates which control for both Hit and False rates. Akin to the other two tasks measuring giving up, overall giving up scores were measured as the percentage of uncertain responses out of all possible face selections (180).	See Wilhelm et al. (2014) and Figure A2 in the Appendix A.	.89 (Law et al. 2018)
**Cognitive tasks with embedded confidence ratings**		
*Esoteric Analogies Test (EAT)—Stankov (1997)*. The EAT is a measure of both fluid and crystallised intelligence with the current study using a twenty-item version. For each item, participants are shown a pair of words and an additional word. They are then given four multiple choice options and must decide which option relates to the additional word in the same way that the pair of words relate to each other. Accuracy on this score is measured by the percentage of items correct.	SPACE is to POINT as TIME is to: CLOCK, ETERNITY, MOMENT*, POSITION	.70 to .72 (Kleitman et al. 2018; Stankov 1997)
*Berlin Numeracy Test (BNT)—Cokely et al. (2012)*. The BNT is a measure of numerical ability, more specifically, statistical and risk literacy. We used the four-item version of the BNT, with every item being a mathematical question which asks for an open-ended numeric answer. Accuracy was measured through percentage of items correct. Although the measure only consists of four items, it has been shown to predict risk understanding more effectively than other longer numeric and intelligence measures.	Imagine we are throwing a five-sided die 50 times. On average, out of these 50 throws how many times would this five-sided die show an odd number (1, 3 or 5)? ____ out of 50 throws	Test-retest reliability: .91 (Cokely et al. 2012).
*Raven’s Advanced Progressive Matrices (RAPM)—Raven (1938) and Waschl et al. (2016)*. RAPM is a measure of fluid intelligence which has been used frequently within the individual differences literature (McGrew 2009; Waschl et al. 2016). For each item, participants are shown a 3 × 3 grid of abstract figures with the bottom right figure being left blank. The participants are shown eight different options and must decide which option best fits the pattern shown in the grid. Based on previous research, the current study used a 15-item version which has been validated in prior research (Waschl et al. 2016). Accuracy is measured as the percentage of correct items.	See Raven (1938) and Figure A3 in the Appendix A for example.	.80 (Jackson et al. 2017)
*Cognitive Reflection Test (CRT)—Frederick (2005) and Toplak et al. (2014)*. The CRT is a reasoning measure of susceptibility to heuristics and biases where participants must answer open-ended questions which have been constructed to prompt incorrect intuitive answers. The current study used the extended 7-item CRT (Toplak et al. 2014). Accuracy on the CRT is measured by percentage correct.	A bat and a ball cost $1.10 in total. The bat costs a dollar more than the ball. How much does the ball cost? ____ cents.	.70 (Jackson et al. 2016)
*Applying Decision Rules (ADR)—(de Bruin et al. 2007).* The ADR is a reasoning measure testing heuristics and biases susceptibility where participants are shown attribute information on five different DVD players, with four different attributes (e.g., sound quality) each with varying levels of performance ratings from 1 (very low) to 5 (very high). For each of the ten items, participants are given a scenario and must then select the DVD player/s which best fit the scenario. An accuracy score is computed by the percentage of answers correct.	LaToya only wants a DVD player that has got a “Very High” rating on Sound Quality	.60 (Jackson et al. 2016)
**Decision-Making Styles**		
*General Decision-Making Styles (GDMS) Scale—(Scott and Bruce 1995).* The GDMS scale measures people’s decision-making tendencies with 25 self-report items with 5 items each relating to 5 different decision-making styles: rational, avoidant, dependent, intuitive, and spontaneous. Participants must respond from 1 (strongly disagree) to 5 (strongly agree) for each item. Average scores for each decision-making style are calculated, with higher scores indicating higher style propensity.	Rational: I make decisions in a logical and systematic way. Avoidant: I avoid making important decisions until the pressure is on. Dependent: I rarely make important decisions without consulting other people. Intuitive: When making decisions, I rely upon my instincts. Spontaneous: I often make impulsive decisions	.67 to .87 (Scott and Bruce 1995; Spicer and Sadler-Smith 2005)
**Real-Life Outcomes**		
*Academic performance.* To measure academic performance, overall weighted average marks (WAM) from consenting participants were collected (n = 130).	-	-

^a^ Initial testing used four-second time periods as delays for incorrect response of (n = 25). This was changed to eight seconds following participant feedback, suggesting that four-second delays were insufficient/not noticeable. No significant differences were found between participants with the different levels of delays however, and thus, results were examined together for analyses, t(*174*) = −1.78, *p* = .08 for accuracy and t(*174*) = −1.04, *p* = .31 for giving up scores.

**Table 2:** Descriptive statistics, reliabilities, and correlations between metacognitive and cognitive variables.

	Mean	SD	1	2	3	4	5	6	7	8	9	10	11	12	13	14	15	16
**Giving Up Score**																		
1. SUD	4.61	7.01	(.94)															
2. CUBE	14.02	15.35	.32 **	(.91)														
3. VS	3.73	3.45	.21 **	.03	(.78)													
**Confidence**																		
4. EAT	68.96	11.22	.02	−.21 **	.06	(.83)												
5. BNT	63.38	24.93	.07	−.17 *	.06	.29 **	(.83)											
6. RAPM	65.60	19.93	−.01	−.16 *	.05	.46 **	.46 **	(.91)										
7. CRT	76.70	17.77	.00	−.29 **	.03	.47 **	.54 **	.52 **	(.79)									
8. ADR	83.65	14.69	.02	−.22 **	.02	.53 **	.41 **	.57 **	.61 **	(.87)								
**Performance Accuracy**																		
9. SUD	87.17	3.80	.20 **	.07	.16 *	−.01	−.02	−.02	−.04	−.02	(.79)							
10. CUBE	74.55	13.39	.14	.10	.07	.24 **	.36 **	.36 **	.17 *	.21 **	.02	(.85)						
11. VS	70.61	12.10	−.01	−.05	.00	.01	.11	.05	−.04	−.10	.07	.12	(.86)					
12. EAT	64.13	16.06	.09	−.05	.11	.43 **	.25 **	.30 **	.30 **	.29 **	.02	.36 **	.15 *	(.69)				
13. BNT	42.61	30.39	.16 *	−.07	.12	.17 *	.52 **	.29 **	.31 **	.19 *	.09	.30 **	.18 *	.39 **	(.55)			
14. RAPM	60.68	22.75	.05	.02	.12	.23 **	.38 **	.69 **	.30 **	.30 **	.06	.52 **	.10	.37 **	.39 **	(.79)		
15. CRT	46.43	32.16	.11	−.04	.15 *	.27 **	.50 **	.40 **	.45 **	.31 **	−.02	.44 **	.13	.39 **	.53 **	.49 **	(.78)	
16. ADR	62.69	21.55	.11	−.09	.05	.21 **	.36 **	.23 **	.19 *	.37 **	.01	.44 **	.12	.43 **	.38 **	.38 **	.50 **	(.64)

Note: * *p* < .05, ** *p* < .01; SUD—Sparse Uncertainty Dense, CUBE—Cube Comparisons, VS—Visual Search, EAT—Esoteric Analogies Test, BNT—Berlin Numeracy Test, RAPM—Raven’s Advanced Progressive Matrices, CRT—Cognitive Reflection Test, ADR—Applying Decision Rules. (Cronbach’s alpha estimates) are shown in the diagonal for all variables. Descriptive statistics (mean and SD) are given as %s.

**Table 3:** Model description and fit statistics for CFA with all giving up, confidence, and accuracy variables.

	χ^2^	df	χ^2^/df	TLI	CFI	RMSEA (90% CI)	AIC	SRMR
One-factor model	387.502	104	3.726	.618	.669	.124 (.111–.138)	22,370.5	.092
Two-factor model	309.666	103	3.006	.719	.759	.107 (.093–.121)	22,294.7	.084
Three-factor model	284.124	101	2.813	.746	.786	.101 (.088–.116)	22,273.1	.080
Four-factor model	277.246	98	2.829	.744	.791	.102 (.088–.116)	22,272.2	.079
Three-factor model—Modified	123.273	95	1.298	.958	.967	.041 (.015–.060)	22,124.3	.060

χ^2^—Chi-squared, df—degrees of freedom, TLI—Tucker–Lewis Index, CFI—Comparative Fit Index, RMSEA—Root Mean Square Error of Approximation, CI—Confidence Interval, AIC—Akaike Information Criterion, SRMR—Standardized Root Mean Squared Residual.

**Table 4:** Correlations between the giving up, confidence, and cognitive ability factors with decision-making styles and academic performance.

	Giving Up	Confidence	Cognitive Ability
GDMS Avoidant	.05	−.15 *	−.03
GDMS Dependent	.08	−.01	.00
GDMS Intuitive	−.11	−.08	−.16 *
GDMS Rational	.18 *	.20 **	.10
GDMS Spontaneous	.01	.05	.02
Academic Performance	.19 *	.18 *	.35 **

GDMS—General Decision-Making Style, ** *p* < .01, * *p* < .05.

**Figure A1:**
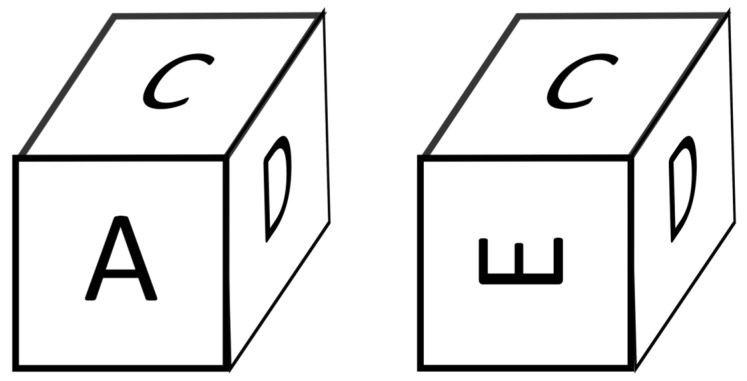
Pseudo-example item for the Cube Comparisons Task. In this example, the two cubes must be different, as the A and E occupy the same position (below the C, and to the left of the D), and any letter can only appear once on each cube.

**Figure A2:**
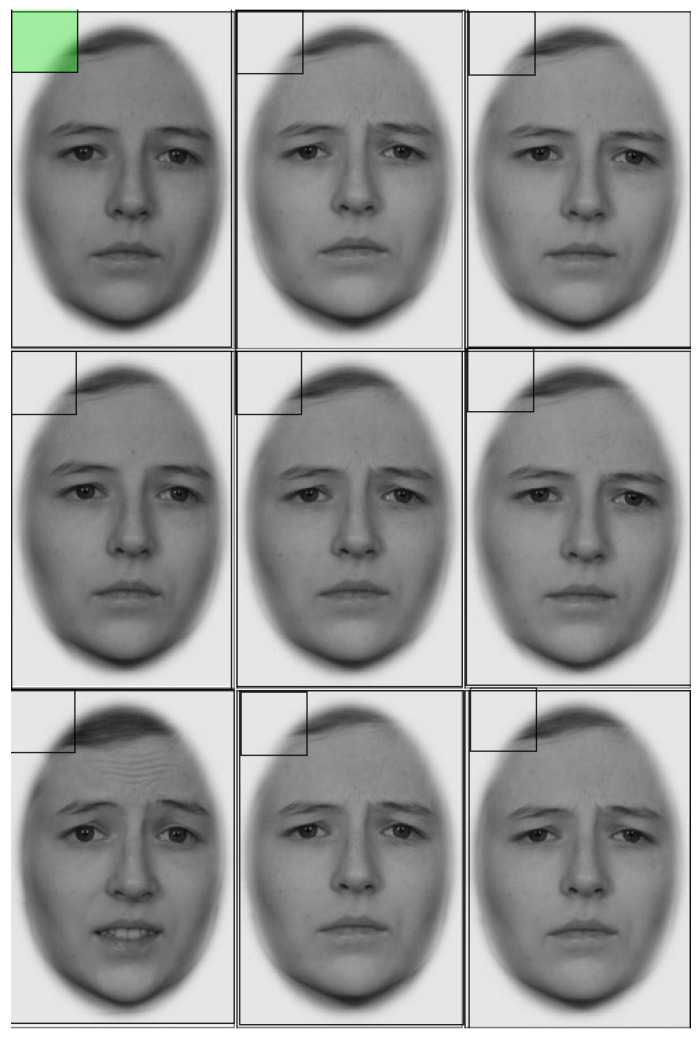
Example item for the Visual Search task. In this example, there are eight faces indicating sadness and one indicating disgust. The top left corner of the top left face was selected, indicating uncertainty towards whether the face displayed the majority emotion or not.

**Figure A3:**
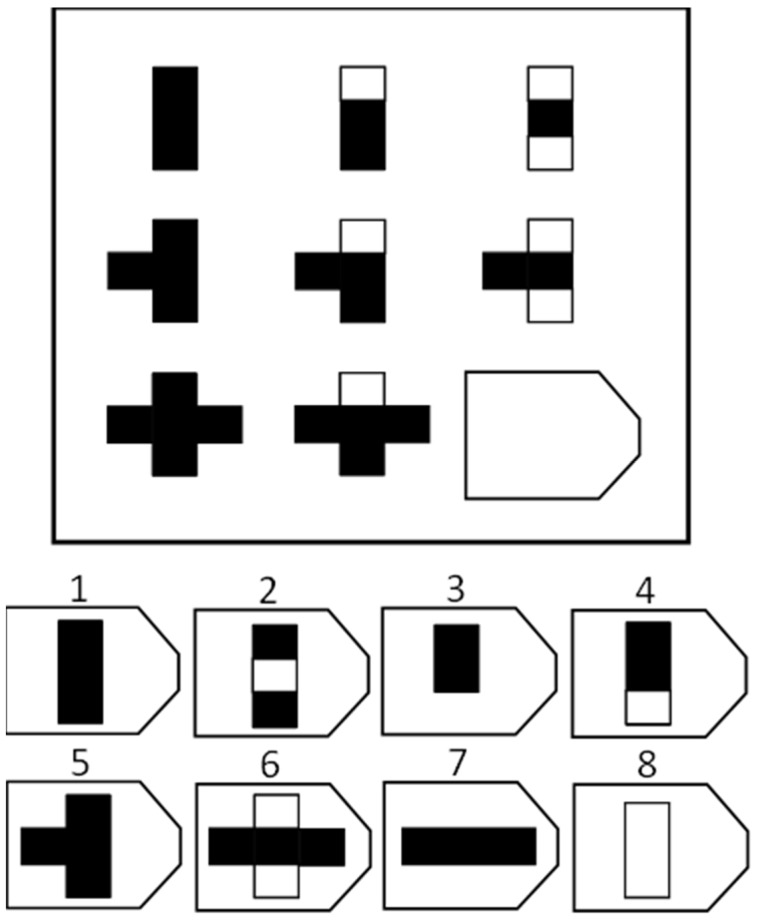
Pseudo-example item for the Raven’s Advanced Progressive Matrices. In the example, the current answer is pattern 6, as it fits both the vertical and horizontal patterns.

## Data Availability

The data presented in this study are openly available from the OSF repository at DOI: 10.17605/OSF.IO/5NK8R.

## Notes

1 Whilst the animal psychology literature labels this paradigm as “uncertainty monitoring”, this terminology may be confusing due to the metacognitive and decision-making literatures’ use of certainty judgements (i.e., confidence). In such fields, uncertainty is treated synonymously as the antithesis of certainty (e.g., 0% confidence on a response being correct). Thus, to increase clarity, we refer to the “uncertainty monitoring” variables measured in the current study as “giving up” behaviour to align with terminology which is more commonly used in the cognitive and decision-making literature.

2 When factor analyses were conducted with the paid participants removed, the pattern of results was analogous to the full sample analyses. Thus, both samples were combined to improve power (analyses conducted without paid participants can be provided upon request from the corresponding author).

## Appendix A

**Table A1:** Descriptive statistics and reliabilities for decision-making variables and academic performance.

	Mean	SD	α
GDMS Avoidant	3.15	0.89	.87
GDMS Dependent	3.82	0.65	.75
GDMS Intuitive	3.64	0.61	.76
GDMS Rational	3.86	0.59	.75
GDMS Spontaneous	2.90	0.69	.80
Academic Performance	70.77	9.88	-

GDMS—General Decision-Making Styles.

**Table A2:** Descriptive statistics, reliabilities, and correlations with factor scores of all other measured variables.

	Mean	SD	α	Correlations		
				Giving Up	Confidence	Cognitive Ability
**Executive Function**						
Flanker Difference Score	0.10	0.09	-	−.05	−.15	**−.16 ***
Task Switching Difference Score	0.08	0.15	-	−.10	−.01	−.05
Running Letters Accuracy	0.47	0.19	.71	**.15 ***	**.26 ****	**.38 ****
**Heuristics and Biases**						
Risky Gambles	0.42	0.17	.66	.09	.08	.04
Resistance to Framing	36.18	4.85	.62	.10	.06	.09
Social Norms Rank Score	0.51	0.22	.37	.00	.07	**.16 ***
Consistency in Risk Perception	0.87	0.12	.51	.00	**.18 ***	.12
**Personality**						
Mini-IPIP Extraversion	3.05	1.01	.87	−.01	−.07	**−.15 ***
Mini-IPIP Agreeableness	4.00	0.72	.72	.03	−.13	−.14
Mini-IPIP Conscientiousness	3.22	0.81	.68	.10	.04	−.06
Mini-IPIP Neuroticism	3.09	0.81	.65	−.06	**−.17 ***	−.03
Mini-IPIP Intellect	3.86	0.69	.69	.03	.06	.11
BAS Drive	2.72	0.59	.80	−.01	.10	−.13
BAS Fun Seeking	3.00	0.54	.70	−.05	.03	−.10
BAS Reward Responsiveness	3.39	0.42	.72	−.10	−.02	**−.25 ****
BIS	3.15	0.51	.79	−.01	−.10	−.07
Need for Closure	3.83	0.64	.82	−.05	.03	−.02
**Other Variables**						
Medical Decision-Making Test Accuracy	0.49	0.24	.78	.01	**.15 ***	**.34 ****
Adapted Decision Outcomes Inventory Score	−0.15	0.08	-	−.03	.14	.13

Note: * *p* < .05, ** *p* < .01.
